# How to improve collaboration between the public health sector and other policy sectors to reduce health inequalities? – A study in sixteen municipalities in the Netherlands

**DOI:** 10.1186/s12939-016-0384-y

**Published:** 2016-06-22

**Authors:** Ilse Storm, Frank den Hertog, Hans van Oers, Albertine J. Schuit

**Affiliations:** National Institute for Public Health and the Environment, Centre for Health and Society, PO Box 1, 3720 BA Bilthoven, The Netherlands; Tilburg University, Tranzo Scientific Center for Care and Welfare, PO 90153, 5000 LE Tilburg, The Netherlands; National Institute for Public Health and the Environment, Executive Office, PO Box 1, 3720 BA Bilthoven, The Netherlands; Department of Health Sciences and EMGO Institute for Health and Care Research, VU University Amsterdam, De Boelelaan 1105, 1081 HV Amsterdam, The Netherlands; National Institute for Public Health and the Environment, Centre for Nutrition, Prevention and Health Services, PO Box 1, 3720 BA Bilthoven, The Netherlands

**Keywords:** Health inequalities, Intersectoral collaboration, Health in All Policies (HiAP), Public health, Physical sectors, Social sectors

## Abstract

**Background:**

The causes of health inequalities are complex. For the reduction of health inequalities, intersectoral collaboration between the public health sector and both social policy sectors (e.g. youth affairs, education) and physical policy sectors (e.g. housing, spatial planning) is essential, but in local practice difficult to realize. The aim of this study was to examine the collaboration between the sectors in question more closely and to identify opportunities for improvement.

**Method:**

A qualitative descriptive analysis of five aspects of collaboration within sixteen Dutch municipalities was performed to examine the collaboration between the public health sector and other policy sectors: 1) involvement of the sectors in the public health policy network, 2) harmonisation of objectives, 3) use of policies by the relevant sectors, 4) formalised collaboration, and 5) previous experience. Empirical data on these collaboration aspects were collected based on document analysis, questionnaires and interviews.

**Results:**

The study found that the policy workers of social sectors were more involved in the public health network and more frequently supported the objectives in the field of health inequality reduction. Both social policy sectors and physical policy sectors used policies and activities to reduce health inequalities. More is done to influence the determinants of health inequality through policies aimed at lifestyle and social setting than through policies aimed at socioeconomic factors and the physical environment. Where the physical policy sectors are involved in the public health network, the collaboration follows a very similar pattern as with the social policy sectors. All sectors recognise the importance of good relationships, positive experiences, a common interest in working together and coordinated mechanisms.

**Conclusion:**

This study shows that there is scope for improving collaboration in the field of health inequality reduction between the public health sector and both social policy sectors and physical policy sectors. Ways in which improvement could be realised include involving physical policy sectors in the network, pursuing widely supported policy goals, making balanced efforts to influence determinants of health inequalities, and increasing the emphasis on a programmatic approach.

## Background

### What is the problem?

Health inequalities are often linked to inequalities in various other areas, such as income, employment, educational level, living and working conditions, lifestyle, and accessibility of care services [1, 2]. Therefore, health inequalities are seen as a complex health problem. In the Netherlands, average life expectancy amongst people with a low level of education is six years less than that amongst people with a high level of education. The difference in life expectancy in perceived good health is even nineteen years [3]. In order to tackle health inequalities, it is important that actions aimed at the underlying determinants involve not only the public health sector but also social policy sectors (e.g. youth care, education) and physical policy sectors (e.g. housing, spatial planning) [4]. Addressing such complex health problems requires ‘health in all policies’ (HiAP) [5–8]. HiAP is a horizontal complementary policy-related strategy with a high potential for contributing to public health [9, 10]. It is assumed that collaboration between policy sectors inside and outside the public health domain is an important precondition for the establishment and implementation of HiAP [11, 12]. The importance of HiAP is stressed in several Dutch national policy documents and programmes, such as the national health policy document *Health Close to people* and the national prevention programme *Everything is Health* [13–15]. Consequently, the reduction of health inequalities by means of HiAP is on the agenda within an increasing number of Dutch municipalities [16]. However, in practice a significant challenge remains to collaborate between the public health sector and both social and physical sectors [17–19].

### What is known and not known?

Within the Netherlands, there are major discrepancies amongst municipalities in terms of the degree of collaboration on complex health problems between the public health sector and other policy sectors [16, 19]. Research has shown that collaboration – and therefore HiAP – is not easy to realise, due to differences in the objectives of and the interests that exist within the various policy sectors [11, 20]. It has also been shown that the development of a joint approach and collaboration through networks (e.g. multidisciplinary teams) are very difficult to achieve [21]. Collaboration may, in fact, be the reason why HiAP has progressed so slowly [21]. Furthermore, while action to influence health outcomes is undertaken -intentional or unintentional- by policy sectors other than the public health sector, it does not automatically follow that there is also collaboration on a complementary policy-related strategy (i.e. multi-sectoral policy collaboration) [8, 22]. With regard to the reduction of health inequalities, policies and interventions were more directed at the individual than the environment, and therefore not all relevant sectors involved [1, 17, 18]. In order to promote coherent approaches to tackling health inequalities at the local level, a national support programme *Health in the city* was launched in summer 2014 [4]. This programme covers one of the goals of the national prevention programme *Everything is Health* for 2014–2016 [15]. Despite knowledge on intersectoral collaboration is more often available and the importance is addressed in national support programs, we have little knowledge of extent to which – and ways in which –the public health sector collaborates with the social policy sectors and the physical policy sectors to reduce health inequalities in Dutch local practice.

### What the study will address?

A previous study of sixteen Dutch municipalities found that the public health sector – usually acting as the initiator of health inequality policy – collaborates with social policy sectors on the reduction of health inequalities more often than with physical policy sectors [16]. Other studies show similar results in the field of complex health problems [23, 24]. The aim of the study reported here was to look more closely at the collaboration between the public health sector’s and both the social policy sectors (e.g. youth affairs, education, sport) and the physical policy sectors (e.g. housing, spatial planning, environment) within the same sixteen municipalities, and to identify opportunities for improvement. The improvement of (this) intersectoral collaboration will, in the end, contribute to a HIAP approach. Although collaboration with other external actors, such as schools, businesses, care organisations, and private citizens, is also necessary for a coherent approach to tackling health inequalities. However, collaboration with such actors is outside the scope of this study. Hence, this study is concerned only with municipal policy sectors.

## Method

### Five aspects of collaboration used as a basis

The public health sector’s collaboration with other policy sectors on health inequalities were analysed by reference to five aspects of collaboration that have previously been identified as important for HiAP [25–27]. These are:

1. Involvement of the appropriate policy sectors in the public health policy network
2. Harmonisation of objectives and priorities across the relevant policy sectors
3. Coordinated use of policies and activities by the relevant policy sectors
4. Formalised collaboration amongst the relevant policy sectors
5. Experience of collaboration amongst policy sectors and favourable contextual factors

The five aspects of collaboration listed above were selected in consultation with an internal health inequality working group at the National Institute for Public Health and the Environment (RIVM) and have been further operationalised for this study through assumptions (see also Data analysis).

### Study-design

To compose a sample, municipalities for this study were selected by initially identifying 50 candidate municipalities (out of a total of about four hundred) active in the field of HiAP. The candidates were selected on the basis of information obtained from the Health Care Inspectorate (IGZ) and the association of community health services in the Netherlands (GGD-GHOR NL), who identified the municipalities as active in the field of HiAP by analysing the content of municipal policy documents [28]. In order to obtain a heterogeneous sample, 24 of the 50 municipalities were selected on the basis of the following criteria: size (large/medium-sized/small), presence/absence of deprived neighbourhoods, and geographical distribution. A further criterion was that the municipal health policy document should explicitly identify the reduction of health inequalities as an objective. To recruit these municipalities for participation, health policy workers (at operational level) were telephone accessed by researchers of this study. Of the 24 municipalities thus selected, sixteen proved willing to participate in the study (period 2009–2010). Those health policy workers that declined cited lack of time or interest as their reasons, and were working at large, medium-sized and small municipalities. The participating health policy workers were asked to provide contact details of their colleagues of other policy sectors. These comprised social sectors, like youth affairs, education, social affairs, health care, sport and integration, and physical sectors like, housing, spatial planning, environment, neighbourhoods, safety, and mobility (mainly at the operational level). The policy workers of these sectors were invited to participate. The municipalities differed in terms of the municipal departments responsible for the various sectors of policy (and the titles of the departments in question).

### Data collection

In order to analyse the five aspects of collaboration within sixteen municipalities, data was collected with three different methods: document analysis, digital questionnaires and interviews.

- *Document analysis:* The public health policy documents of the sixteen participating municipalities were used (period 2008–2012).
- *Digital questionnaires:* A total of 155 digital questionnaires were sent to policy workers within the public health sector as well as the social and physical policy sectors (an average of ten per municipality). Of those, 123 were returned, representing a response rate of 79 %. The respondents were mainly policy officers and policy advisors. In a few cases, they were municipal programme managers, policy developers, project leaders or departmental heads. The digital questionnaire was designed for relatively quick completion on the basis of closed answer categories (Yes/No/Don’t know). The questionnaire responses were used to inform research into the study questions only where the respondents explicitly stated that they were involved in collaborating on health inequalities. Hence, 98 of the 123 questionnaires were ultimately included (80 %).
- *Interviews:* A total of 32 semi-structured interviews were conducted. The research team began by interviewing policy workers whose portfolios included public health (sixteen interviewees). The other policy sectors were involved based on the results of these interviews. Interviews were then conducted with municipal staff working in sectors with which the public health policy staff regularly collaborated, namely the social policy sectors of education/youth affairs (four interviewees), sport (four interviewees), and social affairs (four interviewees). Interviews were additionally conducted with staff active within sectors with which the public health policy staff had indicated more collaboration was desirable (for instance in connection with healthy neighbourhoods), namely the physical policy sectors of housing/spatial planning (four interviewees). Each interview lasted about an hour and a half, and was recorded using a digital voice recorder. Interviews were conducted by researchers of the National Institute for Public Health and the Environment in the period June – November 2009.

The questions posed in the questionnaires and interviews regarding the five aspects of collaboration were operationalised to be specific to the reduction of health inequalities and were piloted in two non-participating municipalities (see Data analysis and Appendix 1). The questionnaires and interviews used for the study are itemised in Table 1.

**Table 1:** Breakdown of questionnaires and interviews

Method	Number	Respondent’s sector	Professions respondents
Online questionnaires	14	Public health	policy officer (4x), policy advisor (7x), program/project manager (2x), policy developer (1x)
	6	Education	policy officer (3x), policy advisor (2x), policy developer (1x)
	11	Youth affairs	policy officer (6x), policy advisor (3x), manager (1x), coordinator (1x)
	8	Social affairs	policy officer (4x), policy advisor (2x), manager (2x)
	5	Housing	policy officer (2x), policy advisor (1x), project manager (1x), manager (1x)
	5	Spatial planning	policy advisor (1x), program/project manager (3x), manager (1x)
	10	Sport	policy officer (5x), policy advisor (3x), program manager (1x), manager (1x)
	3	Mobility	policy officer (1x), manager (2x)
	5	Integration	policy advisor (3x), program/project manager (2x)
	4	Environment	policy officer (1x), policy advisor (1x), manager (1x)
	8	Care	policy officer (3x), policy advisor (3x), manager (2x)
	8	Safety	policy officer (3x), policy advisor (3x), coordinator (2x)
	6	Other social^a^	policy officer (2x), policy advisor (1x), policy developer (1x), coordinator (2x)
	5	Other physical^a^	policy developer (1x), program/project manager (2x), manager (1x)
	*(98 in total)*		
Interviews	16	Public health	policy officer (7x), policy advisor (6x), program/project manager (3x)
	4	Youth affairs/Education	policy officers (3x), senior policy advisor (1x)
	4	Social affairs	policy officer (1x), policy advisor (1x), managers (2x)
	4	Sport	policy officer (2x), policy advisor (2x)
	4	Spatial planning/Housing	policy officer (1x), policy developer (1x), manager (1x), policy developer (1x)
	*(32 in total)*		

^a^ For analysis, respondents were divided into social policy staff and physical policy staff (see Data analysis)

Participants who filled in the digital questionnaires or took part in the interviews were informed that their contributions would be anonymously included in the results. They were informed that results would be presented as group results and would not be reducible to individuals. On the basis of these conditions and prior to the execution of the interviews recorded on tape, participants agreed to take part and gave verbal informed consent to use the results in publications.

### Data analysis

The way that each of the five aspects of collaboration important for the establishment and implementation of HiAP in the sixteen municipalities was analysed is described below. See also Appendix 1.

- *Involvement of the policy sectors in the public health policy network (Aspect 1):* The questionnaires addressed whether the public health sector collaborated with other policy sectors (e.g. youth affairs, education, sport, spatial planning, housing) on the reduction of health inequalities, and – if so – with which sectors; the interviews focussed on whether the right sectors were involved and how multi-sectoral collaboration could be (further) improved.
- *Harmonisation of policy objectives and priorities (Aspect 2):* The questionnaires addressed the reduction of health inequalities as a policy priority and the influence from the relevant policy sectors. The interviews focussed on whether policy objectives were harmonised. Public health policy documents were analysed to determine the abstraction level of the objective of reducing health inequalities.
- *Coordinated use of policies and activities (Aspect 3)*: The questionnaires addressed use of policies and activities within the relevant policy sectors aimed at determinants (causes) of health inequality, such as low incomes, unemployment, low educational level, poor living and working conditions, unhealthy lifestyles and inaccessibility of care services. The interviews – as well as the analysis of policy documents – focussed on the measures and activities used in practice to reduce (particular) health inequalities.
- *Formalised collaboration (Aspect 4):* The questionnaires addressed the means of collaboration (information exchange; implementation of standalone activities; coordination of activities; pursuit of shared objectives) amongst the relevant policy sectors, whereas the interviews focussed on the preferred means of collaboration as well as practical collaborative arrangements and systems linked to health inequalities.
- *Experience of collaboration and contextual factors (Aspect 5):* The questionnaires addressed the respondents’ experience of collaboration with the relevant policy sectors on the reduction of health inequalities (e.g. existence of good relationships, uniform language use, sufficient support); the interviews focussed on the process of collaboration and the requirements for improving collaboration between the public health sector and other policy sectors.

This analysis of data was qualitative for the policy documents and interviews, and quantitative for the digital questionnaires. The data from the questionnaires were descriptive analysed using SPSS 22.0.

For the purpose of analysing separately the social and physical policy sectors in the questionnaires, the sectors education, youth affairs, social affairs, care, sport, and integration were regarded as belonging to the social policy domain. The sectors housing, spatial planning, environment, safety, and mobility were regarded as belonging to the physical policy domain. In the context of the analysis, the public health sector was regarded as a separate domain. Policy workers were classified on the basis of the sector in their portfolio to which they devoted most hours. See Appendix 2 for more background of municipalities and sectors. The qualitative interpretation of the data was performed by the first author (researcher) and discussed in the research team with other researchers.

## Results

### Involvement of policy sectors in the public health policy network (Aspect 1)

**Fig. 1:**
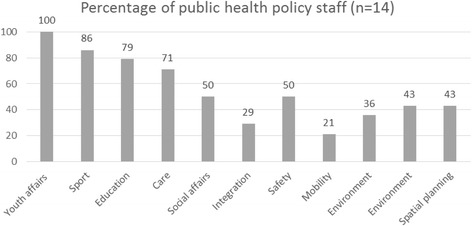
Percentage of public health policy staff who collaborate with other sectors in the public health policy network to reduce health inequalities (n = 14)

Figure 1 based on the questionnaires shows that public health sector policy workers (n = 14) collaborate on the reduction of health inequalities more explicitly with social policy sectors than with physical policy sectors. The sectors with which there is most collaboration are youth affairs, sport, education, and care. For example: 86 % of the public health policy workers collaborate with sport policy staff. One public health sector interviewee said for instance that there was considerable overlap with those sectors, and that it therefore made sense to undertake joint activities. The sectors with which there is least collaboration are mobility, environment, housing, and spatial planning. From interviews with physical policy staff, it is apparent that those sectors sometimes lack awareness of the relevance that their policies have for tackling health inequalities. Policy workers whose portfolios include public health reported wanting more collaboration with physical policy sectors, for example in connection with healthy neighbourhoods (e.g. green areas and playground facilities). It was, for instance, outlined by a policy officer public health that the collaboration with social affairs and sport sectors was more efficiently, but the collaboration with housing and spatial planning sectors improved by shared interest on themes.

### Harmonisation of policy objectives and priorities (Aspect 2)

**Fig. 2:**
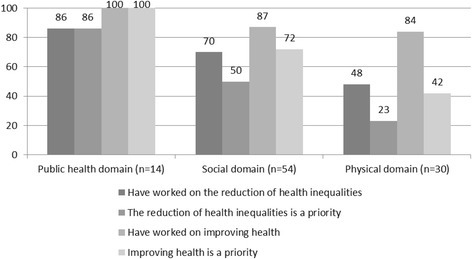
Percentage of policy staff working to address health inequalities (n = 98)

Figure 2 based on the questionnaires shows that, although both physical policy sectors and social policy sectors within the studied municipalities endeavour to improve health, physical policy sectors are involved in the reduction of health inequalities less often than social policy sectors. Moreover, the reduction of health inequalities appears to be a relatively low priority within physical policy sectors as yet. Interviewed policy workers indicated that objectives relating to health inequalities were often defined in abstract or general terms, while those relating to local health issues (overweight, exercise, alcohol, and mental health problems) tended to be more specific. This was confirmed by the analysis of local policy documents. A number of interviewees also reported that the reduction of health inequalities was not (yet) perceived to be a shared responsibility or a widely supported objective. For example, a policy officer spatial planning mentioned that the own sector is working on the realization of an exercise-friendly environment, however this was not done with the aim to contribute to the reduction of health inequalities.

### Coordinated use of policies and activities (Aspect 3)

**Fig. 3:**
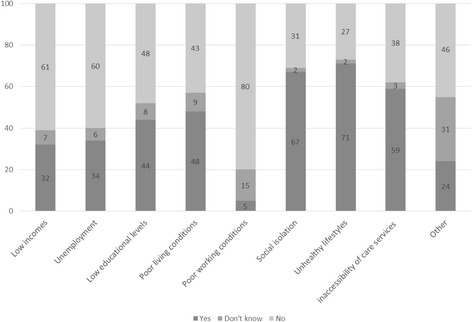
Percentage of policy staff whose policies aimed at determinants (causes) of health inequality (n = 98)

Figure 3 based on the questionnaires shows that when policy workers collaborate on reducing health inequalities, they focus on combatting unhealthy lifestyles, preventing social isolation, and raising the quality of care. There is less attention for poor living conditions, low educational levels, unemployment, and low incomes. A number of municipalities have policies that address several determinants at once (e.g. lifestyle, physical environment, and social setting). Interviewees indicated that the causes of health inequality are varied, and that municipalities consequently addressed various determinants. Table 2 based on the interviews lists examples of the policies and activities deployed in the physical and social policy sectors. It was apparent from the interviews that such activities can contribute to the reduction of health inequalities but are not always intended for that purpose or are presented in coordination with other policies. For instance, a policy officer social affairs outlined that policy not specifically address the reduction of health inequalities, and a policy officer spatial planning stated that the relations between health problems is not always recognized. According to the interviewees, there is scope for more systematic effort to establish a coherent approach. One interviewee said that if there were an underlying programme, it would be easier to identify areas of overlap between sectors.

**Table 2:** Examples of policies and activities that reduce health inequalities (intentionally or otherwise)

Social	Physical
Education policy • Community schools/facilities at schools • Reducing dropout • Community internships • School accommodation • Indoor school environment • Projects aimed at pre-vocational secondary schools (lifestyle, drugs, safe sex, and overweight)	Housing policy • Making houses more sustainable • Improving older districts • Deploying social programmes when houses are renovated in neighbourhoods to be restructured • Report desk for nuisance and problems
Youth policy • Youth and family centres (help with parenting and growing up) •Reducing alcohol and drug use amongst young people •Use of baby clinics for extra consultations in deprived neighbourhoods	Spatial planning policy • Sports facilities and playgrounds • Exercise-friendly environment • Greenery
Social policy • Collective health insurance for people on low incomes • Reimbursement of costs for using municipal facilities •Guiding people into work (incl. the ‘Fit for work’ programme) • Improving the wellbeing of people on benefits	Environmental policy • Noise abatement • Improving indoor environments • UMTS radiation and high-voltage lines • Healthy and clean cities
Care policy • Improving access to care • Reducing social isolation • Health promotion (overweight)	Healthy neighbourhoods • Neighbourhood visions/plans • Sporting neighbourhoods
Sport policy • Local sports clubs •‘Combination officers’ • Providing sports facilities • Sport promotion projects • After-school sport • Encouraging young people from ethnic minorities to participate in sport	Safety policy •Improving the health of repeat offenders • Increased neighbourhood safety for lower-SES residents • Organising help in individual problem situations
Integration policy • Encouraging employment participation amongst ethnic minorities • Improving socio-cultural integration and perception	Mobility policy • Good public transport in deprived neighbourhoods

The examples come from questionnaire and interview responses; the effectiveness of the policies and activities has not been assessed

### Formalised collaboration (Aspect 4)

**Fig. 4:**
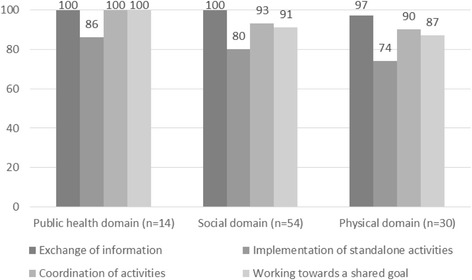
Percentage of policy staff using various collaboration strategies to reduce health inequalities (n = 98)

From Fig. 4 based on the questionnaires, it is apparent that there are no significant differences between the social and physical policy sectors in terms of the way they collaborate on reducing health inequalities. This implies that, once the physical policy sectors are involved in the public health policy network, collaboration will generally take the same form as it does with social policy sectors. In both cases, the strategies are aimed at the exchange of information, the implementation of standalone activities, the coordination of activities, and/or working towards a shared goal. It was apparent from the interviews that, in the early stages of collaboration, the focus was mainly on the exchange of information (informal relationship), while later on, working towards a shared goal (formal relationship) was more common. For example, a policy officer public health mentioned that it is time consuming to get those involved in another mindset at the beginning of the collaboration. Interviewed policy workers also indicated that collaborative arrangements aimed at reducing health inequalities were more likely to concern projects (e.g. lifestyle projects or healthy school/neighbourhood programmes) than policy process (e.g. integrated policy and decision-making). It was for instance outlined by a policy officer sport that they consulted the public health sector regarding shared target groups when developing their own policy document, but no integrated policy making regarding the reduction of health inequalities has taken place. The forms of consultation between sectors vary and are closely linked to the collaboration method, which may take the form of *ad hoc* collaboration, coordination meetings, workgroups, or integrated teams (also known as multidisciplinary teams). One public health policy worker said that the forms of consultation were not always led by health problems, but by the need to identify areas of overlap (e.g. healthy neighbourhoods). Working in integrated teams appears to be the most formal method of intersectoral collaboration aimed at reducing health inequalities. It was apparent from the interviews that the form of consultation did not depend so much on the policy sector, but on the organisation and the vision within the municipality in question (which may already work more systematically in integrated teams).

### Experience of collaboration and contextual factors (Aspect 5)

**Fig. 5:**
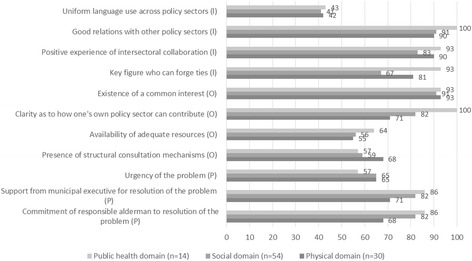
Percentage of policy staff with previous experience and context in collaboration (n = 98)

Figure 5 based on the questionnaires shows that policy staff in the social policy sectors had experiences of collaborating on health inequalities that were broadly similar to the experiences of colleagues working in physical policy sectors. There were nevertheless certain differences as well. Similarities were the existence of good relationships, positive experiences, and a common interest in working together. The experience is that the presence of a key figure who can forge ties has a positive effect. Interviewees from the public health sectors indicated that they often played a coordinating role in bringing sectors together to address complex health problems (for instance acting as process supervisor or programme manager).

It is apparent from the interviews that policy workers regard working with other sectors on an issue as a positive thing, which reinforced each other’s goals. Policy workers also indicated that they got on with each other well, particularly when cooperating on projects. Speaking the same language and having sufficient resources can further enhance collaboration, but in that regard, there were no obvious differences between the social policy sectors and the physical policy sectors. One difference was that it was clearer to staff working in social policy sectors how their policies could contribute to the reduction of health inequalities. From the interviews with the physical policy sector workers, it is apparent that their involvement can be increased by providing them with accessible information about health issues and how these are related to their policy sector. Still, the forms of consultation for collaboration on health inequalities within the physical policy sectors were more structural – which may be a necessary thing. One example is the regular ‘healthy neighbourhood’ consultation meetings attended by representatives of the various sectors. Organisationally speaking, policy workers from the social policy sectors find it easier to interact with public health policy staff, because they often work in the same municipal department. Interviewees indicated that the support of departmental managers and responsible aldermen was essential for intersectoral collaboration on the reduction of health inequalities. It was for instance outlined by a policy officer social affairs that the collaboration between different sectors was more likely to occur when the importance was stressed by the council of the municipality. Within the social policy sectors, the levels of support and commitment from the relevant aldermen are currently greater than within the physical policy sectors.

## Discussion

### Collaboration between the public health sector and other policy sectors

The study reported here examined the extent to which – and ways in which – the public health sector collaborates with both the social policy sector and physical policy sector on the reduction of health inequalities in Dutch practice; it also aimed to identify opportunities for improving collaboration with a view for a better HiAP approach (HiAP). With those aims in mind, a qualitative descriptive analysis of five aspects of collaboration within sixteen Dutch municipalities was performed: the involvement of the appropriate policy sectors in the public health policy network, the harmonisation of objectives, the use of policies by the relevant policy sectors, collaboration methods, and the previous experiences of the policy staff concerned. The opportunities for local policy staff at the operational level to act on collaboration aspects are considered below.

### Opportunities for collaboration in the establishment and implementation of HiAP

The study found that the sectors youth affairs, education, sport and care were more likely to be involved in the public health policy network with a view to reducing health inequalities than the sectors environment, mobility, housing, and spatial planning. That is despite the fact that it is apparent from the literature that the involvement of both social policy sectors and physical policy sectors is vital [29, 30]. Policy staff working in the public health sector also report that there is scope for involving physical policy sectors more in their networks, and thus for establishing and implementing a better HiAP approach. Interaction amongst policy sectors within the public health policy network is important in the context of addressing community issues [24, 29].

The study also found that there is scope for both social policy sectors and physical policy sectors to influence the determinants of health inequality on a more coordinated and deliberate basis, and to pursue more widely supported objectives in the sectors involved. For example, in the interests of a coherent approach with the explicit objective of reducing health inequalities, physical policy sectors could contribute more to improving living conditions (e.g. reducing traffic congestion and creating meeting places), while social policy sectors could contribute more to improving socioeconomic circumstances (e.g. eliminating or reducing language development delay in children) [1, 30]. Naturally, such initiatives should not be at the expense of the continuation of existing efforts to change unhealthy lifestyles, tackle social isolation, and raise care quality standards [31]. The broader determinants serve as good starting points for the establishment of HiAP [32]. Other research in Dutch municipalities found that the determinants of public health were not explicitly used as starting points for policy processes [23, 33]. If a policy problem justifies policy integration beyond action by one sector, it might be helpful to start from the determinants of public health (because of the broad health perspective), to systematically translate them into policy and to define broad policy goals, since that would give sectors the opportunity to participate [33]. To get other sectors involved in collaborative efforts to establish HiAP, it will of course be necessary to consider what the public health sector can contribute to those other sectors, and to look for areas of overlap [8, 34].

The study found that there are also opportunities for (more) formal collaboration on health inequalities and for gaining positive experiences. Collaboration may be regarded as an iterative process [35]. For example, formal collaboration with physical policy sectors could be promoted by demonstrating how such sectors can contribute to influencing (determinants of) health inequalities. Other important requirements for such collaboration are the support of managers and aldermen with responsibility for physical policy sectors [36]. An increasing number of HiAP tools are available, which can help to integrate health issues into the policies of sectors other than public health (e.g. the Health in All Policies checklist) or to gauge the support for health issues present in other sectors (e.g. the Responsive Evaluation of Integrated Action (RIA) method) [37–39]. For example, this method can be used to shed light on the support for addressing health issues that exists within social and physical policy sectors and to further promote collaboration [40, 41]. In addressing health inequalities, the continuous acquisition of experience and continuous learning are vital for the collaboration process. Good relations and experience were found to have a positive influence on collaboration with all sectors in the sixteen municipalities studied. Naturally, collaboration and investment in relationships cost time and energy. A programmatic (i.e. systematic and coherent) approach and a clear coordinating role for the public health sector can facilitate collaboration with physical and social policy sectors and the implementation of HiAP [18, 36].

### Limitations

The study had certain limitations, which should be considered. First, the municipalities were selected on the basis that they were involved in HiAP activities and identified health inequalities as a theme in their public health policy documents. This implies that the results are not representative for all Dutch municipalities. However, the aim was to identify opportunities for improving collaboration (e.g. harmonisation of objectives, coordinated use of policy, involvement of the appropriate sectors in the policy network, formal collaboration, gaining positive experience) and facilitating collaboration amongst policy sectors.

Second, the study looked exclusively at the experiences of policy workers (at the operational level). The experiences of departmental managers (at the tactical level) and responsible aldermen (at the strategic level) were not explicitly addressed, although they are, according to some interviewees vital for the establishment and implementation of integrated health policy [24]. However, the triangle of data collection methods (questionnaires, interviews, and document analysis) did include questions relating to organisational and political factors (e.g. resource availability, lacking support, commitment of aldermen).

A third limitation of the study is that it has been assumed that monosectoral action (action by one sector) is insufficient for the reduction of health inequalities. In practice, such action can in fact be useful and may be more feasible within municipalities. Moreover, collaboration requires the investment of a lot of time and effort. Nevertheless, on the basis of Dutch and international literature, it has been assumed that HiAP (action by multiple sectors) is the optimum strategy for the reduction of health inequalities [29, 32].

Finally, this in-depth study made use of data from 2009–2010. However, health inequalities are persistent, and tackling them requires prolonged effort. Furthermore, the reduction of health inequalities is on the public health agendas of one in three municipalities [42]. The national support programme *Healthy in the City* has shown that the problems described remain topical for municipalities, and that a coordinated approach remains as important as ever [4].

## Conclusion

This study shows that there is scope for improving collaboration in the field of health inequality reduction between the public health sector and both social policy sectors and physical policy sectors. That applies to all five aspects of collaboration examined in this study, namely involvement in policy network, harmonization objectives, coordinated use of policies, formalized collaboration and experiences of collaboration. The study found that the policy workers of social sectors were more involved in the public health network and more frequently supported the objectives. Both social policy sectors and physical policy sectors used policies and activities to reduce health inequalities. More is done to influence the determinants of health inequality through policies aimed at lifestyle and social setting than through policies aimed at socioeconomic factors and the physical environment. Where the physical policy sectors are involved in the public health network, the collaboration follows a very similar pattern as with the social policy sectors. The involvement of physical policy sectors in the network could be improved by making the relationship between policy in those sectors and health inequalities more explicit, and by seeking more support from the responsible aldermen and departmental managers. All sectors recognise the importance of good relationships, positive experiences, a common interest in working together and coordinated mechanisms. Ways in which improvement could be realised include intensifying efforts to involve physical policy sectors in the public health policy network, making more explicit use of determinants at the start of the collaboration process, working towards widely supported policy goals, focussing on formal collaboration strategies based on a programmatic joint approach (usually coordinated by the public health sector), and working to secure adequate support for action at the tactical and strategic level. By mobilising both social and physical policy sectors within the public health policy network and improving collaboration with them, it is possible to facilitate the establishment and implementation of HiAP with the aim of reducing health inequalities.

## Abbreviations

GGD-GHOR NL, The association of Community Health Services - Regional Medical Emergency Preparedness and Planning in the Netherlands; IGZ, Health Care Inspectorate (IGZ); RIVM, National Institute for Public Health and the Environment; VWS, Ministry of Health, Welfare and Sport

## Appendix 1

In the table below, five aspects of collaboration identified as relevant in the literature are operationalised for the reduction of health inequalities (through assumptions). The formulated questions were posed to policy staff working in various sectors within the sixteen participating municipalities (via electronic questionnaires and in-depth interviews). The sixteen participating municipalities all identified the reduction of health inequalities as an objective in their health policy documents.

**Table 3:** Background to the research questions

	Aspect of collaboration	Assumption for operationalisation	Electronic questionnaire questions	In-depth interview questions
1	Involvement of policy sectors in the public health policy network	Health inequalities are reduced if several policy sectors collaborate in addressing such inequalities and/or their determinants.	- Does your policy sector collaborate with other policy sectors on the reduction of health inequalities? - With which policy sectors does your policy sector collaborate on the reduction of health inequalities (education, youth affairs, sport, care, social affairs, integration, housing, spatial planning, mobility, environment, safety)?	- Why do you collaborate with those particular policy sectors on the reduction of health inequalities? - Do you think that the right policy sectors are involved in collaboration on the reduction of health inequalities? - How can other policy sectors be included in collaboration on the reduction of health inequalities?
2	Harmonisation of policy objectives and priorities across the relevant policy sectors	Health inequalities are reduced if such inequalities and/or their determinants have priority in policy sectors and their reduction contributes to a shared objective.	- Is improving public health a priority within your policy sector? - Has your policy sector worked on improving public health in the last year? - Is the reduction of health inequalities a priority within your policy sector? - Has your policy sector worked on reducing health inequalities in the last year?	- Has a shared health inequality reduction objective been formulated? Why, or whynot?
3	Coordinated use of policies and activities by the relevant policy sectors	Health inequalities and/or their determinants are reduced if there is coordinated use of policies and activities across various policy sectors.	- In the last year, has your policy sector worked on improving disadvantageous factors (such as low income, unemployment, low educational levels, poor living conditions, social isolation, unhealthy lifestyles, or poor quality of care)?	- What practical measures and activities has your policy sector initiated with a view to reducing health inequalities? - Are the measures and activities in question specifically intended to reduce health inequalities, or do they have another primary aim?
4	Formalised collaboration amongst policy sectors	Health inequalities are reduced if multiple policy sectors regard the same issues as important and there are formal collaboration strategies relating to those issues.	- What forms does your collaboration with other policy sectors on the reduction of health inequalities take (e.g. exchange of information, implementation of standalone activities, coordination of activities, and working towards a shared goal)?	- What forms of collaboration has your policy sector initiated? What is desirable? - Have definite collaborative arrangements been made with other policy sectors with a view to reducing health inequalities? - What mechanisms are in place for consulting other policy sectors about the reduction of health inequalities?
5	Experience of collaboration amongst policy sectors and favourable contextual factors	Health inequalities are reduced if there is more intensive collaboration amongst policy sectors and if positive factors are favourably influenced.	- Have the following (individual, organisational, and political) factors had a positive influence on collaboration on the reduction of health inequalities: uniform language use, good relationships, positive experience of collaboration, key figure who can forge ties, existence of a common interest, clarity as to how one’s own policy sector can contribute, availability of adequate resources, presence of structural consultation mechanisms, urgency of the problem, support from the municipal executive for resolution of the problem, commitment of the responsible aldermen to resolution of the problem?	- What is your view of the collaborative process amongst policy sectors on the reduction of health inequalities? - How could collaboration on the reduction of health inequalities be improved?

NB 1. Other questions (in addition to those presented here) were posed in the context of the study of the sixteen municipalities. The table includes only those questions that related to collaboration amongst policy sectors in particular

NB 2. The research was concerned with the effectiveness of collaboration on the reduction of health inequalities (process), not with the effectiveness of action to address health inequalities (outcome)

## Appendix 2

**Table 4:** Characteristics of municipalities and policy sectors

			Public Health Sector	Social policy sectors (number of hours per week)							Physical policy sectors (number of hours per week)						
Municipalities^a^	Number of municipalities	Number of inhabitants^b^	Public Health (n = 14)	Education (n = 6)	Youth Affairs(n = 11)	Social Affairs (n = 8)	Care (n = 8)	Sport (n = 10)	Integration (n = 5)	Other (n = 6)	Housing (n = 5)	Safety (n = 8)	Spatial Planning (n = 5)	Mobility (n = 3)	Environment (n = 4)	Other (like neighbourhood) (n = 5)	Number of respondents ^c^
Large with deprived neighbourhoods (>450.000 inhabitants)	3	747.093	32	-	-	-	-	-	-	-	32	-	28	-	25	-	4
		582.951	40	-	16	20	40	36	-	20	-	-	-	-	-	-	6
		475.681	-	-	22	-	-	-	-	-	-	-	-	-	-	-	1
Medium-sized with deprived neighbourhoods (50.000-200.000 inhabitants)	5	118.182	32	-	-	35	30	40	40	30	-	32	-	-	-	32	8
		97.342	-	-	-	-	-	32	-	-	32	32	24	-	-	-	4
		141.211	28	32	-	30	32	32	36	32	-	--		-	-	36	8
		182.489	36	-	24	24	32	36	-	36	4	-	-	-	-	-	7
		143.582	40	32	32	24	-	-	-	-	32	28	32	-	36	-	8
Medium-sized without deprived neighbourhoods (50.000-200.000 inhabitants)	3	116.878	8	-	28	-	28	36	-	24	36	-	-	-	-	-	6
		140.648	36	-	29	-	-	-	-	-	-	36	-	-	18	-	4
		183.270	26	-	24	22	32	25	36	24	-	-	-	32	-	36	9
Small without deprived neighbourhoods (<45.000 inhabitants)	5	44.472	16	32	-	-	24	-	-	-	-	8	26	40	-	32	7
		41.132	24	24	18	-	-	24	40	-	-	34	-	-	-	12	7
		28.022	24	32	32	-	-	30	40	-	-	36	-	-	18	-	7
		32.243	24	26	10	24	36	22	-	-	-	22	-	-	-	-	7
		23.765	19	-	18	24	-	-	-	-	-	-	40	40	-	-	5
Average			28	30	23	25	32	31	38	28	27	27	30	37	24	30	
Total	n = 16																n = 98

^a^A previous study of the sixteen Dutch municipalities discussed the characteristics of these municipalities [16]

^b^Statistics Netherlands (CBS): number of inhabitants in the period of the study (2009–2010)

^c^Number of respondents that stated that they were involved in collaborating on reducing health inequalities
